# Supplementary material for: Reversible fold-switching controls the functional cycle of the antitermination factor RfaH
Source: Nat Commun. 2019 Feb 11;10:702. doi: 10.1038/s41467-019-08567-6 (PMC6370827; doi:10.1038/s41467-019-08567-6)
Supplement: Supplementary file 1 — Supplementary Information [file 41467_2019_8567_MOESM1_ESM.pdf]

Supplementary Information

Reversible fold-switching controls the functional cycle of the antitermination factor RfaH

P. K. Zuber *et al*.

Supplementary Table 1. Diffusion coefficients of RfaH and RfaH-CTD in the absence and presence of *ops*EC.

	D (m^2 s^{-1})
[I,L,V]-RfaH	$1.09 \cdot 10^{-10} \pm 2.46 \cdot 10^{-13}$
[I,L,V]-RfaH-CTD	$1,53 \cdot 10^{-10} \pm 7.35 \cdot 10^{-13}$
[I,L,V]-RfaH:*ops*EC	$4.79 \cdot 10^{-11} \pm 2.37 \cdot 10^{-12}$
[I,L,V]-RfaH-CTD:*ops*EC	$1.36 \cdot 10^{-10} \pm 2.77 \cdot 10^{-12}$

Supplementary Table 2. Oligonucleotides

Name	Sequence (5'-3')	Source
Fw_rfaH_pET19bmod	GGA ATT CCA TAT GCA ATC CTG GTA TTT ACT GTA CTG	Metabion international AG, Planegg/Steinkirchen, Germany
Rv_rfaH_pET19bmod	CGG GAT CCT TAG AGT TTG CGG AAC TCG G	Metabion international AG, Planegg/Steinkirchen, Germany
ops-T DNA	CAC TGG AAG ATC GAA AAA AGC ACG CTA CCG CCC GCG TGG TGG TG	Metabion international AG, Planegg/Steinkirchen, Germany
ops-RNA	UUC UUU GGC GGU AGC GU	Metabion international AG, Planegg/Steinkirchen, Germany
ops-NT DNA	CAC CAC CAC GCG GGC GGT AGC GTG CTT TTT TCG ATC TTC CAG TG	Metabion international AG, Planegg/Steinkirchen, Germany

Supplementary Table 3. Plasmids.

**Name**	**Description**	**Source**
pVS10	P_{T7} promoter– *E. coli rpoA–rpoB–rpoC*^{His6}*–rpoZ*	Ref. [1]
pIA238	*E. coli rfaH* in pET28a	Ref. [2]
pET19bmod_RfaH	*E. coli rfaH* in pET19b (hexahistidine tag followed by TEV protease cleavage site)	This work
pETGB1a_EcrfaH-CTD(101-162)	*E. coli rfaH C-terminal domain* in pETGB1a (hexahistidine tag followed by Gb1 tag and TEV protease cleavage site)	Ref. [3]
pET11a_EcNusG-NTD(1-124)	*E. coli nusG-NTD* in pET11a	Ref. [4]
pGEX_ecoNusE$^{\Delta}$	*E. coli s10*$^{\Delta}$ in pGEX-6P	Ref. [5]
pET29b_ecoNusB	*E. coli nusB* in pET29b	Ref. [6]
pIA349	T7A1 promoter–37-nt U-less region–*E. coli rfaQ ops*	Ref. [2]

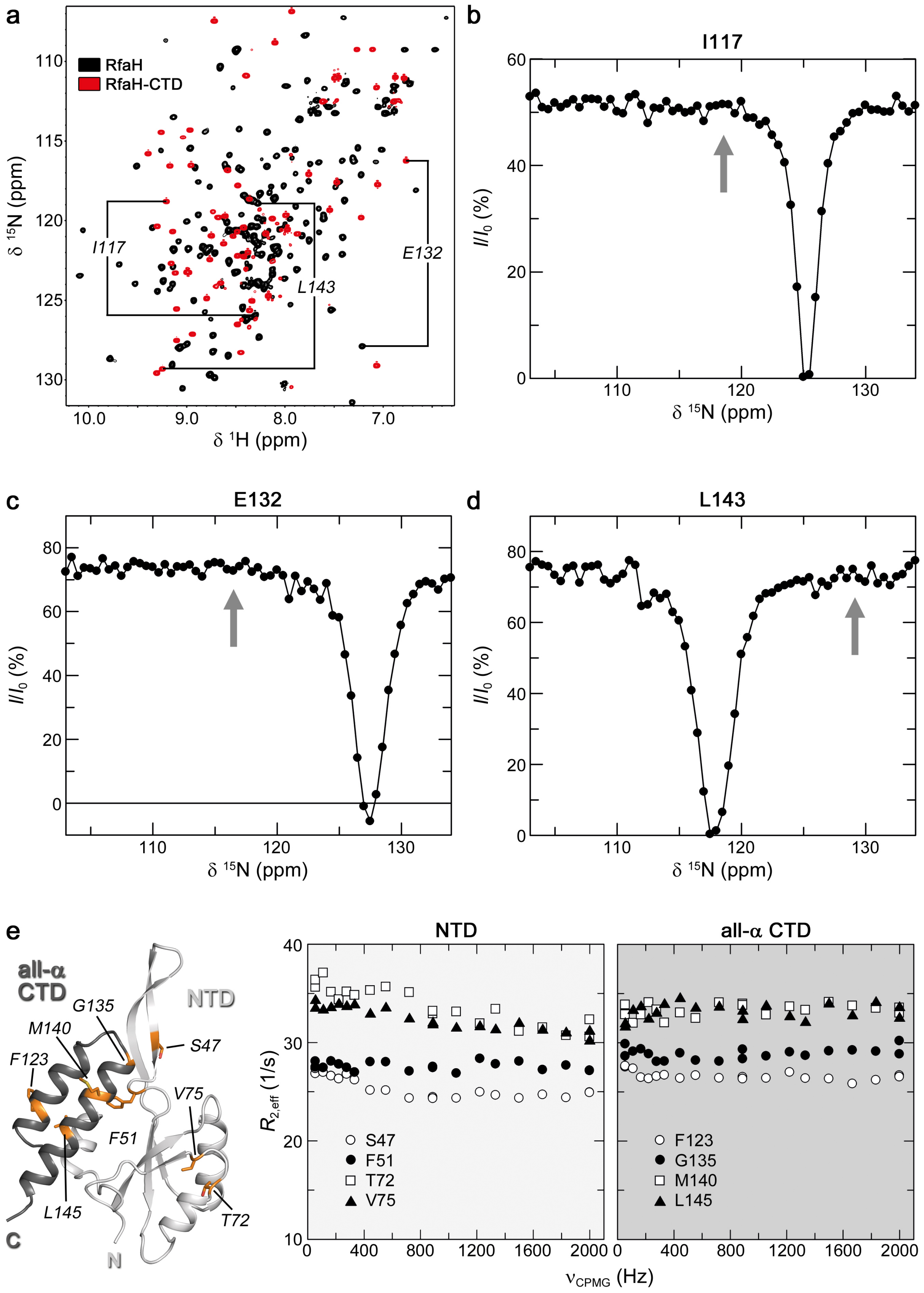

Supplementary Figure 1. The open and closed conformation of RfaH are not in an

equilibrium. **(a)** 2D [^{1}H,^{15}N]-HSQC spectra of 322 μM ^{2}H,^{13}C,^{15}N-RfaH and 100 μM ^{15}N-RfaH-CTD. **(b-d)** ^{15}N-based CEST experiment of ^{2}H,^{13}C,^{15}N-RfaH (322 μM). I/I_0 is plotted against δ(^{15}N) for **(b)** I117, **(c)** E132, and **(d)** L143. Arrows indicate δ(^{15}N) of the corresponding signal in the all-β state. **(e)** ^{15}N-based CPMG experiment of ^{2}H,^{15}N-RfaH (320 μM). (Left) RfaH (PDB ID: 5OND) in ribbon representation (RfaH-NTD, light grey; RfaH-CTD, dark grey). Selected amino acids are shown as orange sticks and labeled. (Right) $R_{2,\mathrm{eff}}$ is plotted against ν_{CPMG} for RfaH-NTD (left panel) and RfaH-CTD (right panel) residues highlighted in the structure.

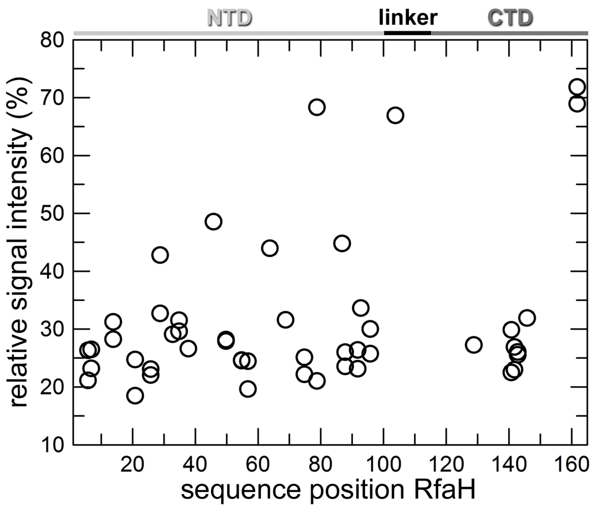

Supplementary Figure 2. Binding of RfaH to RNAP. Titration of [I,L,V]-RfaH with RNAP. Relative intensity of [I,L,V]-RfaH methyl groups after addition of equimolar amount of RNAP *vs.* residue number in RfaH as derived from the methyl-TROSY spectra. Arrangement of RfaH domains is indicated above.

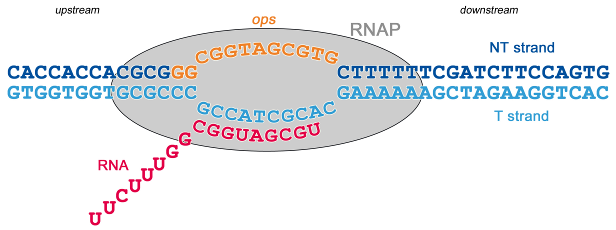

Supplementary Figure 3. The *ops*EC scaffold. The scaffold is designed so that U17 (corresponding to T11 of *ops*) is positioned in the active site. For details on the assembly see Methods section.

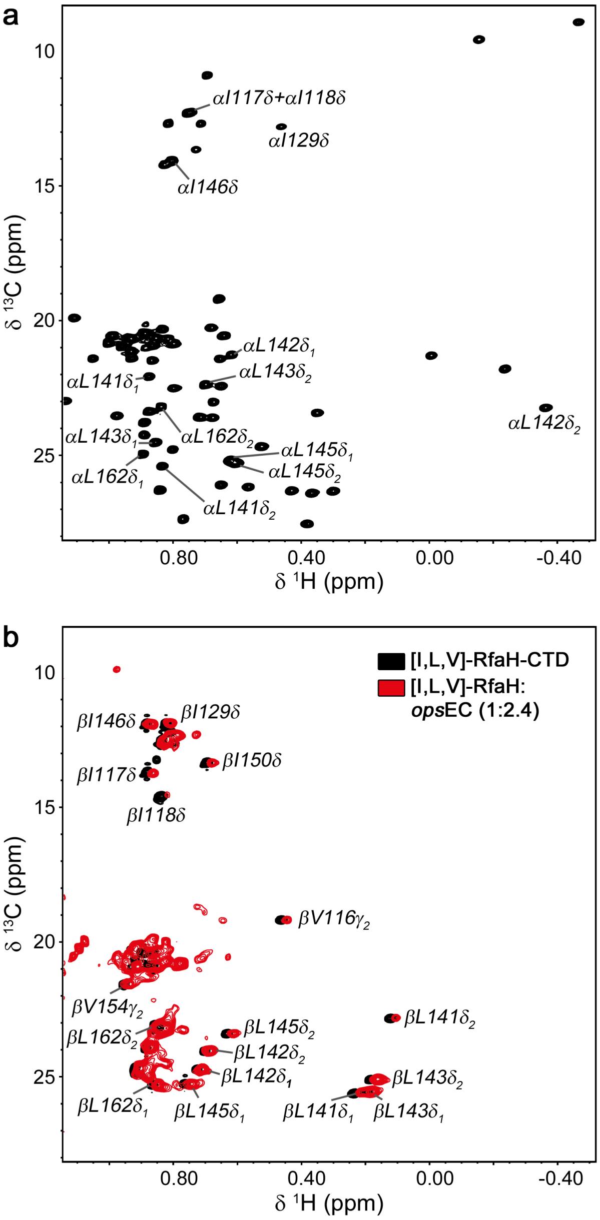

Supplementary Figure 4. Assignment of RfaH-CTD residues in free and *ops*EC-bound RfaH. (a) 2D [^{1}H, ^{13}C] methyl TROSY spectrum of 45 µM [I,L,V]-RfaH. Signals corresponding to RfaH-CTD residues in the all-α state are labeled. **(b)** Superposition of 2D [^{1}H, ^{13}C] methyl TROSY spectra of 80 µM [I,L,V]-RfaH-CTD and 15 µM [I,L,V]-RfaH in the presence *ops*EC (molar ratio 1:2.4). Signals corresponding to RfaH-CTD residues in the all-β state are labeled.

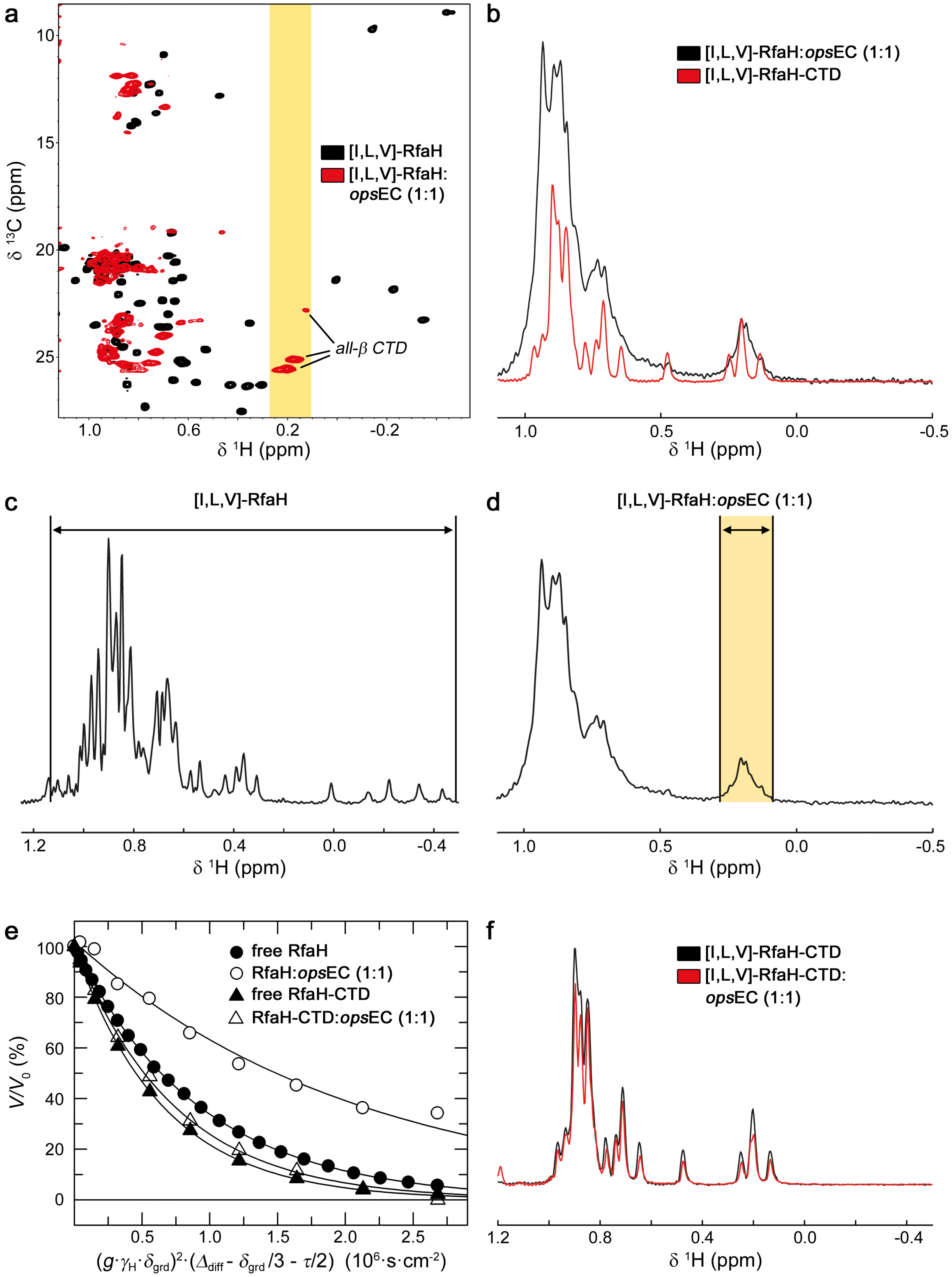

Supplementary Figure 5. Binding of RfaH and RfaH-CTD to *ops*EC. (a) 2D methyl-TROSY spectra of free (black, 50 µM) and *ops*EC bound (red, 38 µM each) [I,L,V]-RfaH. The yellow bar indicates a spectral window containing only all-β RfaH-CTD signals. **(b)** 1D methyl-TROSY spectra of [I,L,V]-RfaH in the presence of *ops*EC (38 µM each, black) and of

80 μM [I,L,V]-RfaH-CTD. **(c,d)** 1D methyl-TROSY spectra of **(c)** free (50 μM) and **(d)** *ops*EC-bound (38 μM each) [I,L,V]-RfaH. Horizontal arrows indicate the regions used for integration in the analysis of the translational diffusion experiments. For [I,L,V]-RfaH:*ops*EC this region corresponds to the spectral window highlighted in **(a)**. **(e)** Determination of the translational diffusion by ^{13}C-edited STE experiments ([I,L,V]-RfaH: 50 μM; [I,L,V]-RfaH:*ops*EC: 38 μM each; [I,L,V]-RfaH-CTD: 100 μM; [I,L,V]-RfaH-CTD:*ops*EC: 40 μM each). Each data set was fitted to a single exponential decay function. The translational diffusion measurements show that all-β CTD signals in the [I,L,V]-RfaH:*ops*EC sample result from RfaH that is bound to the *ops*EC. **(f)** RfaH-CTD binds weakly to the *ops*EC. 1D methyl-TROSY spectra of [I,L,V]-RfaH-CTD in the absence (black; 80 μM) or presence (red, molar ratio 1:1; 40 μM each) of *ops*EC.

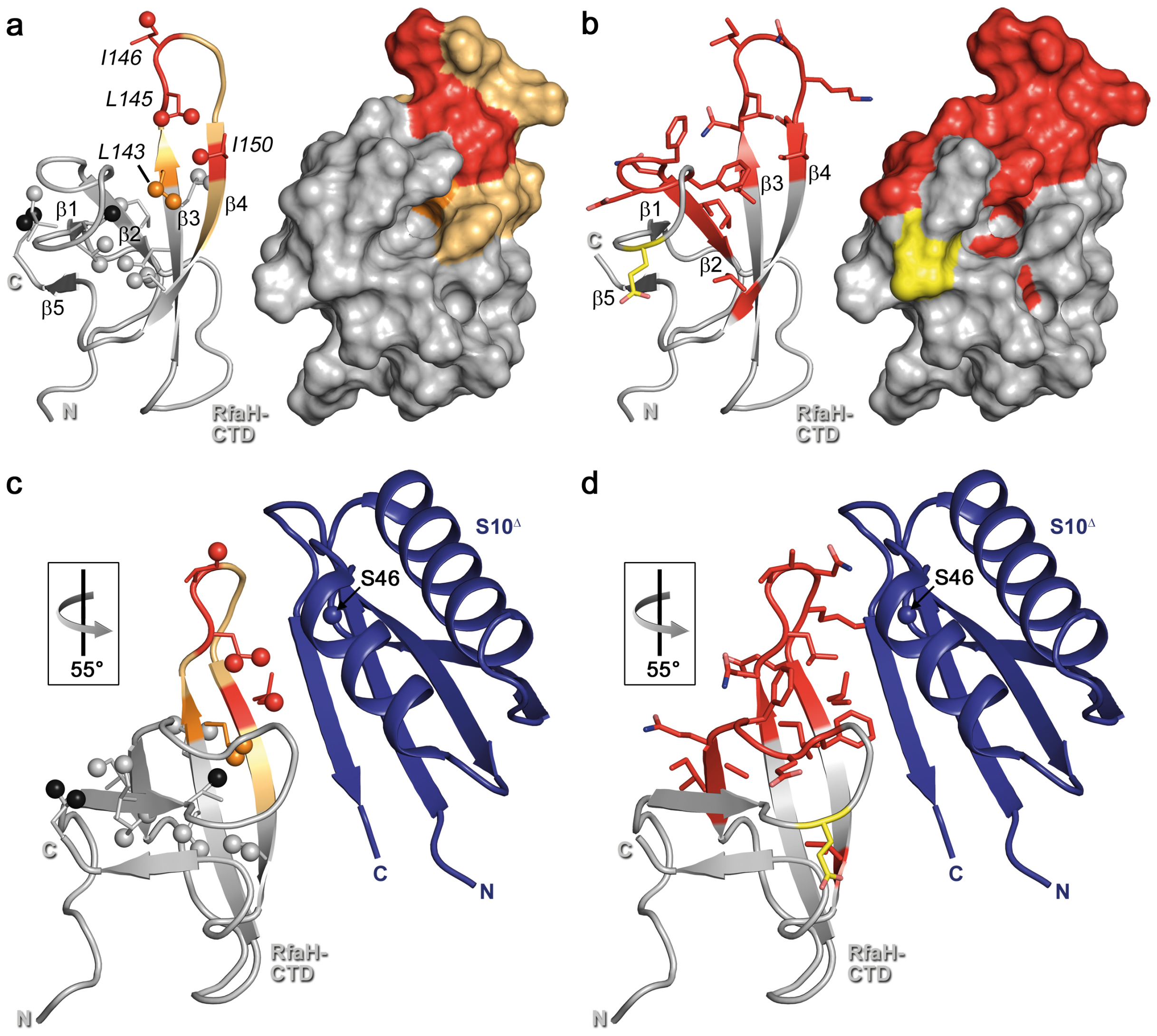

Supplementary Figure 6. *ops*EC-bound RfaH and free RfaH-CTD interact similarly with S10. (a) Mapping of methyl groups of RfaH-CTD affected by binding to S10$^{\Delta}$:NusB while being in complex with *ops*EC as determined by the [I,L,V]-based titration (spectra in Figure 5a). RfaH-CTD (PDB ID: 2LCL; grey) is shown in ribbon (left) and surface (right) representation. For graphical representation of the interaction site the whole amino acid is colored. Ile, Leu, and Val residues are in stick representation with the carbon atom of the methyl groups as sphere. Moderately affected, orange; strongly affected methyl groups, red; unaffected, grey; not assigned methyl groups, black. Termini are labeled. Two amino acids on either side of an affected Ile/Leu/Val residue are highlighted in beige unless they were unaffected Ile/Leu/Val residues. **(b)** Mapping of RfaH-CTD residues affected moderately

(orange) and strongly (red) by binding to S10$^{\Delta}$:NusB as determined by [^{1}H,^{15}N]-based titration using isolated RfaH-CTD and S10$^{\Delta}$:NusB. RfaH-CTD (PDB ID: 2LCL; grey) is in ribbon (left) and surface (right) representation (data taken from Ref. [6]). Affected residues are shown as sticks with nitrogen in blue and oxygen in light red. Secondary structure elements and termini are labeled. **(c,d)** Model of the RfaH-CTD:S10$^{\Delta}$ complex based on the NusG-CTD:S10$^{\Delta}$ complex (PDB ID 3D3B). S10$^{\Delta}$ in ribbon representation (blue). Ser46 replacing the ribosome binding loop is depicted as sphere. Representation of RfaH-CTD as in **(a)** or **(b)**, respectively. The orientation of RfaH-CTD relative to **(a,b)** is indicated.

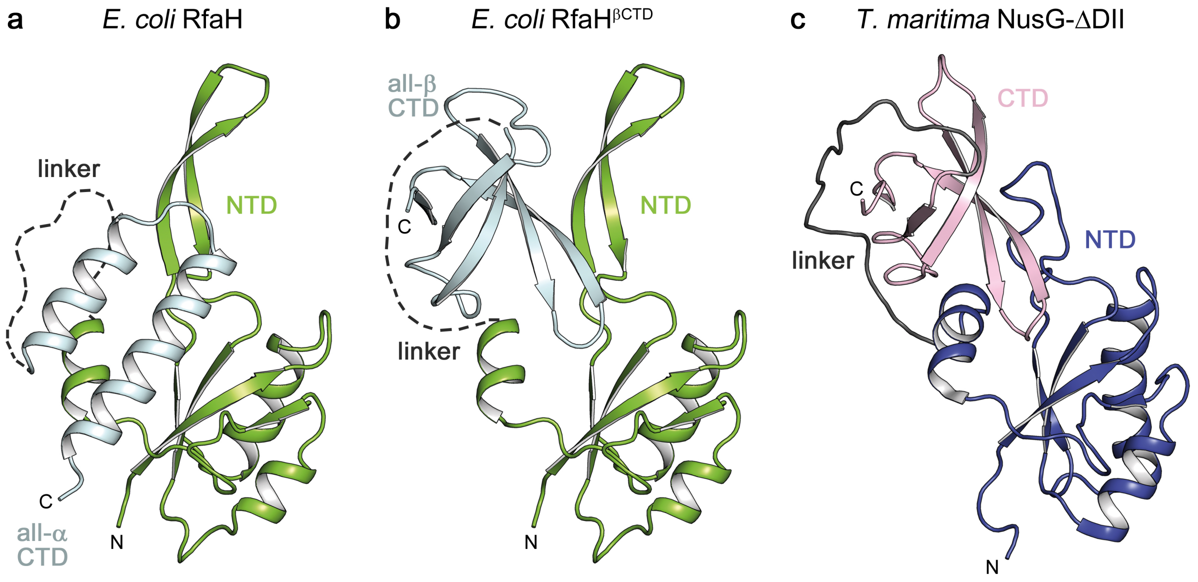

Supplementary Figure 7. Autoinhibition in RfaH and TmNusG. Structures of **(a)** RfaH, **(b)** RfaH modeled in a closed state according to *Tm*NusG with its CTD in the all-β state, and **(c)** *Tm*NusG-ΔDII, a variant of *Tm*NusG where the additional domain DII is deleted, in ribbon representation. PDB IDs: RfaH, 5OND, RfaH-CTD all-β, 2LCL; TmNusG, 2LQ8.

Supplementary References

1. Belogurov, G. A. *et al.* Structural basis for converting a general transcription factor into an operon-specific virulence regulator. *Mol. Cell* **26**, 117–129 (2007).

2. Artsimovitch, I. & Landick, R. The transcriptional regulator RfaH stimulates RNA chain synthesis after recruitment to elongation complexes by the exposed nontemplate DNA strand. *Cell* **109**, 193–203 (2002).

3. Burmann, B. M. *et al.* An α helix to β barrel domain switch transforms the transcription factor RfaH into a translation factor. *Cell* **150**, 291–303 (2012).

4. Burmann, B. M., Scheckenhofer, U., Schweimer, K. & Rösch, P. Domain interactions of the transcription-translation coupling factor *Escherichia coli* NusG are intermolecular and transient. *Biochem. J.* **435**, 783–789 (2011).

5. Luo, X. *et al.* Structural and functional analysis of the *E. coli* NusB-S10 transcription antitermination complex. *Mol. Cell* **32**, 791–802 (2008).

6. Burmann, B. M., Luo, X., Rösch, P., Wahl, M. C. & Gottesman, M. E. Fine tuning of the *E. coli* NusB:NusE complex affinity to BoxA RNA is required for processive antitermination. *Nucleic Acids Res.* **38**, 314–326 (2010).
